# Extracellular vesicles in hepatocellular carcinoma: unraveling immunological mechanisms for enhanced diagnosis and overcoming drug resistance

**DOI:** 10.3389/fimmu.2024.1485628

**Published:** 2024-10-28

**Authors:** Lanqian Su, Yuxin Yue, Yalan Yan, Jianming Sun, Lanxin Meng, Jiaan Lu, Lanyue Zhang, Jie Liu, Hao Chi, Sinian Liu, Zhongqiu Yang, Xiaowei Tang

**Affiliations:** ^1^ School of Clinical Medicine, The Affiliated Hospital of Southwest Medical University, Luzhou, China; ^2^ Department of Pediatrics, Southwest Medical University, Luzhou, China; ^3^ Department of General Surgery, Dazhou Central Hospital, Dazhou, China; ^4^ Department of Pathology, Xichong People’s Hospital, Nanchong, China; ^5^ Department of Gastroenterology, The Affiliated Hospital of Southwest Medical University, Luzhou, China

**Keywords:** extracellular vesicles, hepatocellular carcinoma, tumor marker, diagnosis, drug resistance

## Abstract

Current research is focused on utilizing EVs as a biopsy tool to improve the diagnostic accuracy of HCC, reduce surgical risk, and explore their potential in modulating drug resistance and advancing immunotherapeutic strategies. Extracellular vesicles (EVs) have been increasingly recognized as important non-invasive biomarkers in hepatocellular carcinoma (HCC) due to the presence of a variety of biomolecules within them, such as proteins and RNAs, etc. EVs play a key role in the early detection, diagnosis, treatment, and prognostic monitoring of HCC. These vesicles influence the development of HCC and therapeutic response in a variety of ways, including influencing the tumor microenvironment, modulating drug resistance, and participating in immune regulatory mechanisms. In addition, specific molecules such as miRNAs and specific proteins in EVs are regarded as potential markers for monitoring treatment response and recurrence of HCC, which have certain research space and development prospects. In this paper, we summarize the aspects of EVs as HCC diagnostic and drug resistance markers, and also discuss the questions that may be faced in the development of EVs as markers.

## 1 Introduction

As a non-invasive biomarker, extracellular vesicles (EVs) contain a variety of biomolecules that may play an important role in the early detection diagnosis, precise treatment, and prognostic monitoring of HCC (1–3). The adoption of EV as a biomarker for HCC may improve diagnostic accuracy, enhance therapeutic efficacy, and improve prognostic outcomes (4). Currently, such innovative markers have attracted extensive scientific attention (5–8).

**Figure 1:**
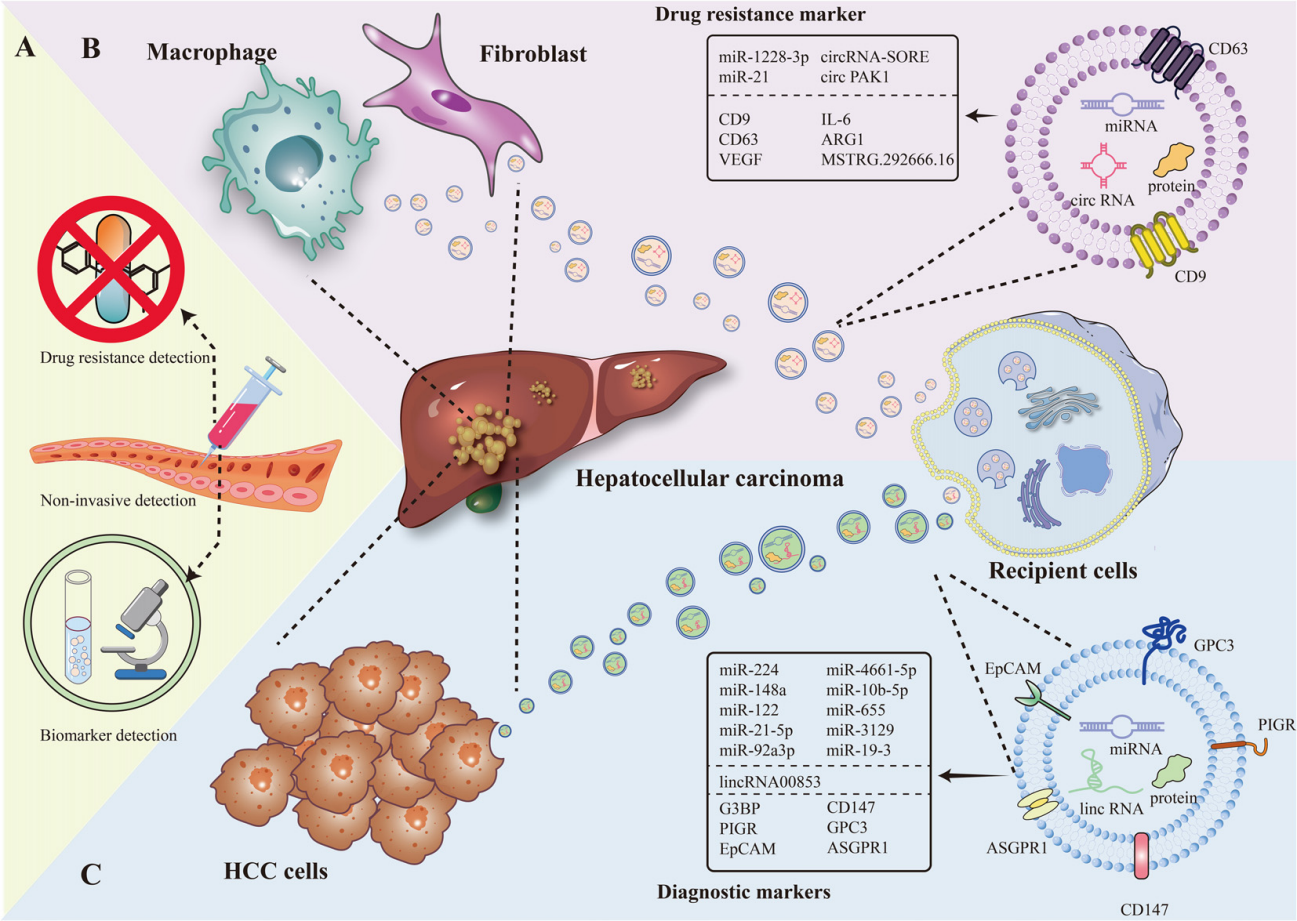
The figure present EV as a marker for diagnosis and drug resistance in HCC in terms of non-invasive testing, EV of tumor cell origin, and EV of immune cell origin. **(A)** Diagnostic markers and resistance markers by non-invasive testing. **(B)** Macrophage, fibroblast-derived EV as drug resistance markers in HCC. **(C)** Hepatocellular carcinoma cell-derived EV as a diagnostic marker in HCC.

This is a heterogeneous group of lipid bilayer boundary particles actively secreted by cells into their surroundings (9), which are divided into three main subtypes: microvesicles/extranuclear granular bodies (Ectosomes) (100-500 nm), Exosomes (30-150 nm), and Apoptotic Bodies (500-2000 nm) (10). They are carriers of a wide range of bioactive molecules originating from the mother cell, including lipids (phosphatidylserine, etc.), RNAs (long non-coding RNAs, mRNAs, etc.), carbohydrates (glycoproteins, glycolipids), and proteins (growth factors, tumor suppressors, enzymes, membrane-integrating proteins, etc.) (11). They are widely distributed in various biological fluids with different sizes, cellular sources of secretion, biological functions and release pathways (12). Meanwhile, EVs play an important role in controlling the tumor microenvironment (TME), and they can control the development of certain cancers, immune escape, angiogenesis, tumor metastasis, proliferation and migration, etc. (**Figure 1A**).

EV secretion is an emerging mechanism by which tumor cells communicate with their surroundings (13). In a variety of cancers, EV can transfer a variety of molecular to the target organ, creating a microenvironment conducive to tumor cell colonization and growth by regulating local cell function, inducing the transformation of mesenchymal cells into fibroblasts (CAF) with pro-tumorigenic properties or directly transforming resident cells into tumor-supportive cells (14, 15). In HCC, EVs can be derived from tumor cells versus non-tumor cells. Since EVs can carry different molecules, and these biomarkers can provide specific information on tumor type, subtype, genetic variation, and drug sensitivity, and the secretion, composition, and functional status of EVs change dynamically with tumor progression, metastasis, the development of drug resistance, and other pathological processes, EVs can be used as a carrier of biomarkers for liver cancer (7, 16).

Through the technical means of liquid biopsy, DNA and RNA in EVs with the help of finer molecular profiling (17), can provide valuable information about early detection, therapeutic monitoring and prognostic assessment of hepatocellular carcinoma (HCC) (18). Exosomes carry genetic information consistent with their parent cells, including DNA fragments, mRNA, miRNA, lncRNA, and others. Detection and analysis of these molecular cargoes provide new methods for non-invasive and precise tumor diagnosis. For example, some experiments have shown that DNA carried by exosomes can be examined by digital PCR (dPCR) or next-generation sequencing (NGS), and that the gene mutation profile of cells can be recognized in the presence of low DNA concentrations (19).

## 2 EV as a biomarker for the diagnosis of HCC

In the past, extracellular vesicles (EV) have (20, 21) become another novel marker for diagnosis by virtue of its special composition (e.g., carrying various types of biomolecules including DNA, RNA, metabolic substances, etc.) and biological functions (involved in inter-cellular communication and cancer progression, etc.) (22). Compared with the low positive rate of traditional direct detection of mi-RNA and protein, EV for diagnosis has more natural advantages: it is widely distributed and can be detected in the blood and other biological fluids of cancer patients; it carries a wide range of tumor-associated molecules, which can specifically reflect the tumor situation. Moreover, EV can protect the nucleic acids within it from decomposition, and EV is highly correlated with the state of its parental cells and can inherit the expression of parental cell traits (23). In addition, the use of EV as a biopsy tool in the diagnostic process can reduce the risk of cancer cell dissemination and infection (24) (**Figure 1B**).

Some data studies have shown that the area under the curve (AUC) of the summarized subject work characteristics (SROC) is 0.88, which may indicate that EV is highly conservative as an early diagnostic marker (25). Meanwhile, subgroup analysis showed that the use of small EV as a biomarker was more accurate (p < 0.001) in serum-based neurological cancer samples (26). In addition, a team of researchers retrieved a total of 3993 records related to HCC by analyzing relevant literature in various databases and screened 18 studies for diagnostic analysis. The results showed that the combined sensitivity of exosomal miRNA amounted to 0.86, which increased to 0.89 when exosomal RNA was used in combination with AFP for diagnostic purposes (27). Various types of studies have demonstrated the greater diagnostic ability and sensitivity of EV (28).

Extraction and purification techniques for EV have been advancing, and the exosome extraction methods currently in use are ultracentrifugation and total exosome isolation kits, among others (29). In terms of testing technologies, emerging technologies such as nano-flow cytometry and super-resolution microscopy have emerged. The former nano-flow cytometry provides high-throughput characterization of exosomes (30), and the latter ultra-high-resolution microscopy provides high-resolution structural information (31), which has become a more precise and efficient tool for exosome research. The importance of exosomes in the diagnostic field is gradually emerging as the RNA and protein content and classes in exosomes are further investigated using cutting-edge technologies.

### 2.1 RNA sorting

The RNA components expressed by exosomes differ between HCC patients and healthy individuals (32–34). Taking miR-224 as an example, several studies demonstrated that its expression level in HCC tissues was significantly higher than that in normal controls (35), suggesting that miR-224 may serve as a potential biomarker for hepatocellular carcinoma. Further validation revealed that the expression of miR-224-containing exosomes was also higher in HepG2 and SKHEP1 hepatocellular carcinoma cell lines compared with normal controls (35). And this high expression of miR-224 was associated with larger tumor volumes and advanced stages of disease (35). From hepatocellular carcinoma cells to sera of hepatocellular carcinoma patients, in conclusion, differential expression of miR-224-containing exosomes may provide more informative support for early noninvasive diagnosis and prediction of HCC.

The diagnostic performance of exosome-derived miRNA as a diagnostic marker for HCC was higher than that of conventional HCC markers. In the HCC group, the area under the curve of miR-148a from serum exosomes was higher than that of alpha-fetoprotein (36). Not only that, some of the mi-RNAs carried by EV had high specificity and sensitivity on HCC. For example, the prediction of HCC using miR-224 had a sensitivity of 92.5%, a specificity of 90%, and an accuracy of 0.94, which was higher than the sensitivity of AFP (37).

In addition to miR-224, there are other EV-carrying miRNAs that have been suggested as biomarkers of early potentiality. For example, miR-21-5p, miR-92a3p, miR-4661-5p, miR-10b-5p, miR-655, miR-3129, miR-19-3 (38–44) and others.

Furthermore, at the level of tumor heterogeneity, RNA and protein cargoes in EVs secreted by different subpopulations of HCC cells reflect tumor suppressive and metastatic potentials, and these molecules reveal phenotypic differences between different subpopulations of HCC cells. Taking miR-122 as an example, it was demonstrated that deletion of miR-122 resulted in increased cell migration and invasion, and conversely, restoration of miR-122 reversed this phenotype (45). This suggests that miR-122 is a marker of hepatocyte-specific differentiation, and that there are differences in the levels of miR-122 in EVs secreted by different subpopulations of HCC cells, which affects tumor suppression and metastatic potential, and can be used to distinguish between different subtypes of HCC.

miRNAs have a clear biological mechanism and a broad research base, however, other RNAs contained within EV, such as highly stable circRNAs and tsRNAs, as well as lincRNAs, which have complex regulatory capabilities and specific expression, have been less studied for possible use as biomarkers. For example, the demonstration of a cohort experiment showed that lincRNA00853 in serum-derived EV showed a high degree of specificity in patients with primary hepatocellular carcinoma (HCC), whereas another scientific team observed differential changes in lincRNA by controlling themselves before and after resection of the tumor (46).Thus, there is an opportunity for lincRNA to be used as a diagnostic marker for early HCC, especially in patients with early-stage tumors who have negative AFP test results (46).

### 2.2 Protein sorting

Previously, the conventional protein-based marker in clinical HCC diagnosis was AFP, which has a sensitivity between 60% and 70% and a specificity of 90% (47). Using a simplified HCC-derived EV surface protein assay (SPA) test, the possibility of utilizing EV-loaded proteins as novel markers was identified, which possessed higher sensitivity and specificity, providing new ideas for the use of proteins in diagnosis (48). For example, tumor cells derived from hepatocellular carcinoma secreted elevated levels of RasGAP SH3 structural domain binding protein (G3BP) and polymeric immunoglobulin receptor (PIGR) contained in EVs. In addition, proteomic profiles constructed by mass spectrometry also demonstrated differential expression of a variety of proteins between different groups (49). These differential expressions provide a basis for the use of protein-carrying EVs as biomarkers. In addition, some EV piggybacked proteins can be used to differentiate early HCC from cirrhosis, including EpCAM, CD147, GPC3 and ASGPR1 (48).

## 3 EV from different cellular sources

Systemic drugs including multikinase inhibitors such as sorafenib and lenvatinib are widely used in the treatment of HCC (3, 50–52). However, after drug application, the dynamics of the cancer cells and the cells in the TME change, and relevant measures are rapidly adopted to relieve the stress generated by cancer treatment. Ultimately this leads to mechanisms of resistance to tumor therapy and provides new therapeutic targets. Tumor cell-derived EV promotes cancer progression by transferring aggressive and drug-resistant phenotypes to other cancer cells (23), and EV is involved in a key metastatic pathway for the development of drug resistance and is a promising liquid biopsy marker, so here we discuss the role of EV produced by various types of cells in HCC tissues in the regulation of drug resistance, and explore whether EV carrying different types of molecules can serve as a mechanism for the development of drug resistance. Therefore, we herein discuss the role of EVs produced by various types of cells in HCC tissues in the regulation of drug resistance, and explore whether EVs carrying different types of molecules can be used as markers for monitoring the development of drug resistance as well as references for precision medicine, in order to provide more strategies for the treatment of HCC.

### 3.1 Mechanisms of HCC drug resistance

Drug resistance in HCC may exist prior to drug administration and can manifest or be enhanced after treatment with the same drug. Although anticancer drugs have multiple targets on HCC, these targets are affected by genetic polymorphisms and heterogeneity, leading to resistance and treatment failure (53). The development of resistance in HCC involves altered cell transduction signaling pathways, dysregulated apoptosis, and the tumor microenvironment (54) (**Table 1**).

**Table 1:** Biomarkers carried by EVs from different parental cell sources.

EV Source	Biomarker	Drug Resistance Receptor Cell	Mechanisms	Effecttion	Reference
M2-TAM	VEGF, IL-6, ARG1	Hepatocellular Carcinoma Cell	Stimulation of vascular growth of tumour tissue, enhancement of tumour invasiveness, suppression of tumour immunity	Promotion	(55–57)
CAF	miR-1228-3p	Hepatocellular Carcinoma Cell	Activation of PLAC8-mediated PI3K/Akt signalling pathway	Promotion	(58)
Hepatocellular Carcinoma Cell	miR-21	Normal Hepatic Stellate Cell	Down-regulation of oncogene PTEN and up-regulation of PI3K/AKT	Promotion	(59)
Hepatocellular Carcinoma Cell	circRNA-SORE	Specific Sensitive Hepatocellular Carcinoma Cells	Blocking PRP19-catalysed breakdown of the key cancer protein YBX1	Promotion	(60)
Hepatocellular Carcinoma Cell	circPAK1	Hepatocellular Carcinoma Cell	Inhibition of the Hippo signalling pathway	Promotion	(61)
HepG2 Cell	miR-774	Low Level MiR-744 Level Cells	Targeted PAX2	Inhibition	(62)
Hepatocellular Carcinoma Cell	CD9, CD63	Hepatocellular Carcinoma Cell	Activation of the HGF/c-Met/Akt signalling pathway	Promotion	(63)
M1-TAM	–	Hepatocyte	Blunt targeting of hepatocyte sensitivity	Promotion	(64)

Typical cellular pathways include the PI3K/AKT pathway (65), MAP/ERK pathway (66), and others. Alterations in these pathways simultaneously activate cancer cell survival and dysregulation of apoptosis. Taking the PI3K/AKT pathway as an example, activation of AKT by PI3K promotes cell survival by inhibiting programmed cell death. At the same time, AKT helps cancer cells avoid death in the presence of drug-induced apoptosis by inactivating pro-apoptotic factors (67).When EV-carried factors activate the PI3K/AKT/mTOR pathway, drug-resistant cells are less sensitive to apoptosis induced by sorafenib action, and in these drug-resistant cells, phosphorylated AKT, and tumor suppressor phosphatases are down-regulated, leading to drug resistance (68). The tumor microenvironment consists of cells, extracellular matrix, and signaling molecules. Immune cells release EVs representing the expression of parental cellular molecules, which exert an immunomodulatory function on the disease progression process. After the action of sorafenib, the redevelopment of HCC is promoted, and the mechanism of redevelopment is related to tumor-associated neutrophil infiltration and the release of cytokines (69). On the one hand, they exacerbate tumor inflammation by driving angiogenesis (70), remodeling the extracellular matrix, and suppressing the immune response. On the other hand, they exert antitumor effects by directly attacking tumor cells or by modulating drug-resistant cell networks of antitumor cells (71).

### 3.2 Tumor cells and mesenchymal cells

#### 3.2.1 RNA sorting

Numerous studies have shown that in HCC tissues, different types of cells release exosomes (EVs) that are piggybacked with specific RNAs, and this leads to a degree of resistance to multikinase inhibitors (72, 73) (**Figure 1C**).

EVs derived from tumor-associated fibroblasts (CAF) also enhance the resistance of HCC to sorafenib treatment. On the one hand, these fibroblasts, after being activated by HCC cell-derived EVs carrying miR-21, produced CAF-EVs containing miR-1228-3p, which enhanced HCC resistance by targeting PLAC8 and activating the PI3K/Akt signaling pathway (58). HCC cells containing miR - 21, on the other hand, the source of EVs by converting normal hepatic stellate cells to CAF, cut tumor suppressor gene PTEN, which make the PI3K/AKT signaling pathway increases (59). Activation of the AKT signaling pathway inhibits autophagy, leading to acquired resistance to sorafenib, a critical step in miR-21-mediated resistance to sorafenib in HCC patients (74).

In addition, cancer cells that have developed drug resistance also produce corresponding EVs to further regulate the development of drug resistance. Taking sorafenib-resistant cells as an example, the EVs produced by them contain circRNA-SORE. circRNA-SORE is transmitted to specific sensitive cells, which can prevent the breakdown of YBX1, a key cancer protein catalyzed by PRP19, and then inhibit the activation of related downstream factors (e.g., AKT, Raf1, ERK, etc.), thus spreading the resistance generated by chemotherapeutic drugs. And a research team demonstrated that silencing circRNA-SORE by injecting siRNA can greatly overcome sorafenib resistance (60). Similarly, lenvatinib-resistant cells induce drug resistance in recipient cells via EV-delivered circPAK1, which works by inhibiting the Hippo signaling pathway, the role of Hippo channels in inhibiting cell growth, proliferation, promoting apoptosis and regulating hepatocytes is critical for tumor suppression, and dysregulation of the Hippo signaling pathway leads to uncontrolled cell proliferation (75). Therefore, it promotes the resistance of receptor cells to lenvatinib drug (61). The above process also demonstrates the transfer of drug resistance from drug-resistant cancer cells to other cancer cells at the level of extracellular vesicles.

#### 3.2.2 Protein sorting

Studies have shown that exosomes containing protein factors can modulate drug resistance in hepatocellular carcinoma (HCC) by activating specific signaling pathways.

Researchers explored the role of exosomes in sorafenib resistance using a subcutaneous tumor transplantation model in thymus-free mice. They found that exosomes enriched with CD9 and CD63 markers promoted resistance to sorafenib by activating the HGF/c-Met/Akt signaling pathway and inhibiting sorafenib-induced apoptosis, enhancing the manifestation of resistance (63). Activation of the HGF/c-Met axis is also one of the causes of resistance to Renvastinib in patients (76).Activation of this pathway resulted in the following resistance effects: HGF attenuated the anti-proliferative, pro-apoptotic, and anti-invasive functions of lenvatinib in HCC cells with high c-MET expression (77). It can be hypothesized that if the activation status of the HGF/c-Met/Akt pathway is monitored in the clinic, the potential resistance of patients to sorafenib or lenvatinib can be predicted, leading to the development of individualized and precise treatment strategies.

### 3.3 Tumor cells and immune cells

The tumor microenvironment (TME) is complex and evolving. In addition to stromal cells, fibroblasts and endothelial cells, the TME includes innate and adaptive immune cells. There is a causal relationship between these immune cells and hepatocellular carcinoma (78). Mechanistically, cytokines within the TME manipulate immune function, ultimately leading to a diminished immune response that directs tumor progression (79). Tumor cell-derived EVs and immune cell-derived EVs are able to interact with each other and together play a role in tumor cell resistance (80, 81). For example, it was experimentally demonstrated that miR-21 transfer carried by tumor-associated macrophage-derived EVs conferred drug resistance in gastric cancer ce.

#### 3.3.1 Macrophage-derived EVs

Macrophage-derived EVs have multiple functions, depending on the various phenotypes of the parental cells, and carry LncRNAs capable of regulating the tumor microenvironment and participating in tumor pathogenesis (82). Excessive insulin levels were found to be significantly associated with an increased risk of hepatocellular carcinoma (83). Exos in pro-inflammatory M1-like macrophage-derived EVs blunts insulin sensitivity in target cells (64), which may be one of the reasons for the dysfunction of insulin signaling pathway in hepatocytes, thus helping hepatocellular carcinoma cells to build up resistance to drugs associated with insulin-sensitizing therapies.

M2 tumor-associated macrophages (M2-TAM) contribute to tumor development through multiple mechanisms, including stimulation of tumor tissue vascular growth, enhancement of tumor invasiveness, and suppression of tumor immunity (55–57, 84). The above mechanisms are realized through their release of EVs enriched with factors such as VEGF, IL-6, and ARG1 (85).

In particular, EVs released by M2-TAM not only play the role of transmitting information and regulating gene expression in the tumor microenvironment, but also influence the behavior and therapeutic response of cancer cells through complex mechanisms. Cutting from the gene expression perspective, these exosomes affect gene expression in cancer cells through two main pathways (86). One is that EVs released by M2-TAM can increase the levels of specific competing endogenous RNAs (ceRNAs). For example, it was found that M2-TAM-derived EV containing MSTRG.292666.16 could significantly increase the level of miR-6386-5p. ceRNAs indirectly increase the expression of target genes by binding to miRNAs and decreasing their inhibition. It promotes the proliferation and survival of tumor cells, thus enhancing resistance to chemotherapeutic drugs. Secondly, it directly regulates the expression of specific genes to enhance the resistance of tumor cells to chemotherapeutic drugs (87). Using known resistance mechanisms, intervention by targeting components in the context of personalized therapy by monitoring EVs containing STRG.292666.16 can be aligned with the goal of precision therapy (88).

#### 3.3.2 Treg cell-derived EVs

Treg (regulatory T) cells are key mediators of tumor-associated immunosuppression (89). Treg cell-derived EVs may represent a fine-grained intercellular exchange apparatus with the ability to modulate the immune response, thereby creating a tolerogenic microenvironment in a cell-free manner. Mechanisms by which Treg cell-derived EVs may mediate immune response include miRNA-induced gene silencing and surface proteins and enzyme delivery. It was found that secretion of EV by CD8 Treg cells stimulated a significant decrease in CD8 T cell responses and protective anti-tumor immunity, while secretion of exosomes by CD8 Treg cells was also able to suppress DC-induced CD8 cytotoxic T lymphocyte (CTL) responses (90, 91). These vesicles were demonstrated to be able to suppress the immune response, thus enabling tumor cells to develop drug resistance (92, 93).

## 4 Conclusion and prospect

Extracellular vesicles (EVs) are gaining attention from the research community as potential biomarkers for the diagnosis and recurrence detection of hepatocellular carcinoma (HCC) (94). EVs as markers also have certain practical difficulties. For example, in terms of standardization, there is a lack of a unified standardized process for purification and isolation, purification and characterization of EVs, the number of target exosomes required is much higher than can now be efficiently produced, and differences in standards and techniques among different laboratories can lead to poor reproducibility and comparability of results (95). If the problem of poor reproducibility and comparability is to be solved in the future, more precise isolation and identification techniques will need to be developed, and exosomes will need to be more finely characterized by emerging means. In terms of samples, the complexity of clinical samples also increases the difficulty of testing. Serum or plasma contains a large number of non-EV components that may interfere with the isolation and detection of EVs, thus affecting the accuracy of the results (96). When selecting substances carried or embedded in EVs as markers, they will face low abundance of markers, which is difficult to detect and identify (97). The development of internationally recognized standardized protocols would provide some assurance of the reproducibility and consistency of EVs study results. As for drug resistance, the limited understanding of the biological mechanisms of EVs in hepatocellular carcinoma drug resistance limits their development and application as diagnostic markers. More basic studies are needed to go deeper, and these basic studies can be combined with multidisciplinary and multi-omics to delve into the specific mechanisms of the role of EVs in HCC. For example, transcriptomics could be combined to study how mRNAs carried in EVs affect drug resistance by influencing gene expression in recipient cells. Machine learning and neural networks can also be used to construct a diagnostic model for hepatitis B-related hepatocellular carcinoma (98, 99). In addition, immune cell-derived EVs can be used as diagnostic tools and preventive strategies at the level of potential biomarkers, e.g., for the development of vaccines, making targeted drugs, etc. Despite the promising research on EVs as biomarkers for hepatocellular carcinoma, they are still at a relatively early stage, and more large-scale clinical studies are needed to validate their specificity, sensitivity and assess the long-term safety and efficacy of EVs in the treatment of HCC, as well as to establish a more standardized detection method (100).
